# Intravenous Thrombolysis Benefits Mild Stroke Patients With Large-Artery Atherosclerosis but No Tandem Steno-Occlusion

**DOI:** 10.3389/fneur.2020.00340

**Published:** 2020-05-05

**Authors:** Dapeng Wang, Lulu Zhang, Xiaowei Hu, Juehua Zhu, Xiang Tang, Dongxue Ding, Hui Wang, Yan Kong, Xiuying Cai, Longting Lin, Qi Fang

**Affiliations:** ^1^Department of Neurology, First Affliated Hospital of Soochow University, Suzhou, China; ^2^Hunter Medical Research Institute, University of Newcastle, New Lambton, NSW, Australia

**Keywords:** mild stroke, thrombolysis, tandem lesion, artery stenosis, artery occlusion

## Abstract

At present, there is controversy regarding whether thrombolysis is beneficial for patients suffering from a mild stroke. In this study, we therefore sought to determine whether the therapeutic benefit of thrombolysis is dependent upon stroke subtype for those with mild stroke. We conducted a retrospective analysis of data from consecutive mild stroke patients (National Institutes of Health Stroke Scale ≤5) with and without recombinant tissue plasminogen activator (rt-PA) therapy. The TOAST (Trial of Org 10172 in acute stroke treatment) criteria was used to determine stroke subtypes. Patients suffering from large-artery atherosclerosis (LAA) were subdivided based upon whether or not they exhibited tandem steno-occlusion, as defined by the association of a proximal intracranial occlusion and a cervical internal carotid artery lesion (complete occlusion or severe stenosis ≥ 90%). For this study, favorable outcomes at 90 days of onset (modified Rankin Scale Score [mRS] of 0–1) were the primary measured outcome. Three hundred thirty-nine patients were included in the study. For patients with non-LAA, there were not statistically significant improvements in favorable outcomes for rt-PA treatment (*p* = 0.889, 0.929, 0.708; respectively). For patients with LAA, compared with non-treated group, rt-PA-treated patients had a significant in the rate of favorable outcomes at 90 days (82.8 vs. 64.9%; OR 2.59; 95%CI, 1.13–5.92; *P* = 0.024). Among LAA patients exhibiting tandem lesions, favorable outcomes were observed in 66.7% of rt-PA-treated patients, with no significant differences to those observed in untreated patients (OR 1.00; 95%CI, 0.23–4.28; *p* = 1.000). Among LAA patients without tandem lesions, compared with non-treated group, we found that rt-PA treatment was associated with a significant beneficial impact on favorable outcomes after 90 days (64.4 vs. 88.4%; OR 4.20; 95%CI, 1.43–12.30; *p* = 0.009). Our findings suggest that intravenous rt-PA is only beneficial in mild stroke patients with LAA-type strokes that do not exhibit tandem steno-occlusion.

## Introduction

Stroke incidence in China is very high, with ~2.5 million cases annually (1), of which 30% are mild ischemic strokes (2, 3). Mild stroke represents one of the main reasons for not receiving intravenous recombinant tissue plasminogen activator (rt-PA) in time-eligible acute ischemic stroke (AIS) patients (4, 5); this is despite current guidelines which make no formal recommendations on of the use of rt-PA in these patients (6). Cases of mild stroke have been excluded due to contraindications from most randomized thrombolysis trials citing reasons that symptoms were not severe enough to warrant a high-risk treatment intervention; although, these decisions are not support by data (7). It must be noted that there is substantive evidence to suggest that a significant proportion of mild stroke patients has subsequent neurological deterioration (8–10). Further, Prior analyses have additionally suggested that stroke subtype may be an independent predictor of prognosis in mild stroke patients (11, 12). A retrospective analysis indicated that both primary rt-PA and endovascular therapy had better outcome at 90 days than primary conservative therapy in patients with large vessel occlusion in the anterior circulation and low NIHSS score (NIHSS score ≤ 5) (13).

Tandem lesions, defined as a combination of high-grade stenosis or occlusion of the internal carotid artery (ICA) with concomitant occlusion of an intracranial vessel (14, 15), accounted for about 10–15% of AIS due to large-artery atherosclerosis (LAA) (16). The treatment of AIS with tandem lesions remains a unique technical challenge due to the low recanalization rate and poor outcomes after rt-PA (17, 18). None of the studies have focused on mild strokes with tandem lesions.

The aim of our study is to identify the impact of rt-PA treatment in mild stroke patients by stroke subtype and further assess the effect on patients with tandem leisons.

## Methods

### Patients Population

**Figure 1:**
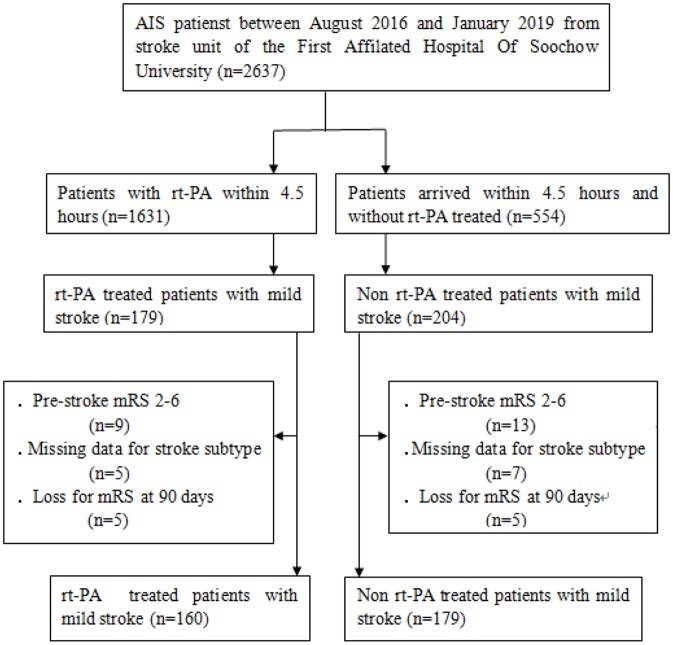
Study inclusion flow chart. AIS, Acute ischemic stroke; mRS, modified Rankin Scale Score.

This study was a retrospective analysis of 339 mild stroke patients (National Institutes of Health Stroke Scale, NIHSS≤5) that had presented in the stroke unit of the First Affiliated Hospital of Soochow University (Figure 1). Patients were included in this analysis if they presented within 4.5 h of stroke onset and either were or were not treated with rt-PA in a standard 0.9 mg/kg dose between August 2016 and January 2019. Inclusion criteria were as follows: (1) patients were NIHSS≤5 and met all other standard IV rt-PA eligibility criteria (19); (2) patients had a modified Rankin Scale (mRS) score ≤1 prior to stroke onset; and (3) patients did not undergo further endovascular therapy within the study period. Patients who had undergone catheter interventions (1), had brain tumors (2), were suffering from severe hepatic or renal dysfunction (3), or were suffering from severe end-stage disease (4) were excluded from analyses. The First Affiliated Hospital of Soochow University ethics committee approved the present study, with all relevant guidelines and regulations being observed. All patients and/or their legal guardians provided informed consent.

Tandem lesions were defined by the association of a proximal intracranial occlusion and a cervical ICA lesion (complete occlusion or severe stenosis ≥ 90%) (20).

The following data were collected for all patients: age, sex, medical history (including smoking status, and history of hyperlipidemia, diabetes, stroke, coronary heart disease, and hypertension), and clinical data collected at time of hospital admission (including stroke severity as measured via NIHSS, onset-to-treatment (OTT) time, stroke subtype, and rate of hemorrhagic transformation). TOAST (Trial of Org 10172 in acute stroke treatment) criteria were used for stroke subtype classification, and LAA strokes were further subdivided based upon whether or not patients were presented with tandem lesions.

### Outcome Measures

The primary outcome for this study was a favorable patient outcome at 90 days, as indicated by an mRS score between 0 and 1 (12, 21, 22). In addition, we analyzed safety outcomes including hemorrhagic transformations (including symptomatic intracranial hemorrhage) based upon ECASS II criteria (23), asymptomatic intracranial hemorrhage, and all-cause mortality within a 90-day period.

### Statistical Analysis

Categorical variables are presented as numbers (percentages), while continuous variables are presented as means [with standard deviations (SD)]. Baseline patient characteristics were compared via Chi-squared (χ2) test, Fisher exact test, Student's *t*-tests, and Mann–Whitney U tests when data were categorical, normally distributed, and non-normally distributed, respectively. A multivariate logistic regression analysis was used to examine factors associated with favorable patient outcomes, following adjustment for specific covariates including age, sex, NIHSS score and OTT time. All tests were 2-sided, and the *p*-value level of significance was set at 0.05. SAS v9.2 (SAS Institute Inc, NC, USA) was used for statistical analyses, and Figures were prepared using GraphPad Prism 5 and Review Manager 5.3.

## Results

### Patient Characteristics

In total, we analyzed outcome data from 160 and 179 patients that were and were not treated via rt-PA, respectively. The rt-PA-treated and -untreated groups had mean ages of 63.4 ± 12.0 and 65.7 ± 12.3 years, respectively (Table 1). There were 44 female patients (27.5%) in the rt-PA treated group and 62 female patients (34.6%) in the rt-PA untreated group. With respect to medical history, in the rt-PA-treated group, 17.5% of patients had a history of stroke, 65.0% had hypertension, 23.1% had hyperlipidemia, 14.4% had atrial fibrillation, and 5.9% had CHD in the rt-PA-treated group, while in the rt-PA-untreated group these percentages were 21.8, 70.9, 20.1, 15.1, and 5.6%, respectively. Patients in rt-PA treated group had a higher proportion of history of smoking (*P* = 0.010), while a lower proportion of history of diabetes mellitus (*P* = 0.029). Laboratory findings were comparable for patients in these two groups, with the exception of LDL-C levels, which were elevated in patients in the rt-PA-treated group relative to untreated controls (2.5 ± 0.8 vs. 2.3 ± 0.8; *P* = 0.018). Patients in the rt-PA-treated and -untreated groups had average NIHSS scores of 2.5 ± 1.4 and 2.2 ± 1.5 at admission, respectively (*P* = 0.066). There was no significant difference in OTT time between the two groups (163.2 ± 57.2 vs. 161.0 ± 57.0 min; *P* = 0.716).

**Table 1:** Baseline characteristics of patients with mild stroke.

**Characteristics**	**rt-PA (*n* = 160)**	**Non-rt-PA (*n* = 179)**	***P***
**Socio-demographic characteristics**			
Female, *n* (%)	44 (27.5)	62 (34.6)	0.157
Age, y, mean ± SD	63.4 ± 12.0	65.7 ± 12.3	0.084
**Clinical status before admission**			
Previous stroke, *n* (%)	28 (17.5)	39 (21.8)	0.322
Hypertension, *n* (%)	104 (65.0)	127 (70.9)	0.241
Diabetes Mellitus, *n* (%)	31 (19.4)	53 (29.6)	0.029
Hyperlipidemia, *n* (%)	37 (23.1)	36 (20.1)	0.500
Atrial fibrillation, *n* (%)	23 (14.4)	27 (15.1)	0.854
Smoking, *n* (%)	54 (33.8)	38 (21.2)	0.010
History of CHD, *n* (%)	10 (5.9)	10 (5.6)	0.796
**Clinical data on admission**			
SBP, mmHg, mean ± SD	151.8 ± 20.7	154.4 ± 22.9	0.276
DBP, mmHg, mean ± SD	83.8 ± 13.4	85.5 ± 14.6	0.264
TC, mmol/l, mean ± SD	4.3 ± 0.9	4.1 ± 1.2	0.108
TG, mmol/l, mean ± SD	1.6 ± 1.0	1.6 ± 1.2	0.804
LDL-C, mmol/l, mean ± SD	2.5 ± 0.8	2.3 ± 0.8	0.018
Cr, umol/l, mean ± SD	73.5 ± 23.0	72.0 ± 25.5	0.571
FPG, mmol/l, mean ± SD	5.9 ± 2.5	6.2 ± 3.6	0.489
NIHSS, mean ± SD	2.5 ± 1.4	2.2 ± 1.5	0.066
OTT time, min, mean ± SD	163.2 ± 57.2	161.0 ± 57.0	0.716
Stroke subtype, *n* (%)			0.553
Large artery atherosclerosis	58 (36.2)	77 (43.0)	
Cardioembolism	29 (18.1)	27 (15.1)	
Small vessel occlusion	65 (40.6)	69 (38.5)	
Other/Undetermined	8 (5.0)	6 (3.4)	
**Intracerebral hemorrhage**			
ASICH, *n* (%)	6 (3.8)	2 (1.12)	0.111
SICH, *n* (%)	2 (1.2)	0 (0.0)	

*SD, standard deviation; CHD, coronary heart disease; SBP, systolic blood pressure; DBP, diastolic blood pressure; TC, total cholesterol; TG, triglycerides; LDL-C, low density lipoprotein cholesterol; Cr, creatinine; FPG, fasting blood glucose; NIHSS, National Institutes of Health Stroke Scale; OTT, onset-to-treatment; ASICH, asymptomatic intracerebral hemorrhage; SICH, symptomatic intracerebral hemorrhage*.

Baseline characteristics of LAA patients with and without tandem lesions were shown in Table 2. There were 33 patients (24.4%) with tandem lesions and 102 patients (75.6%) without tandem lesions. Percentage of diabetes mellitus and hyperlipidemia were higher in the group with tandem lesions (45.4 vs. 23.5%, *P* = 0.016; 24.2 vs. 9.80%, *P* = 0.034). There were no significant differences in baseline characteristics between two groups. Among 102 patients without tandem lesions, 76 patients (74.5%) were with isolated stenosis to occlusion of middle cerebral artery (MCA), 23 patients (22.5%) were with isolated stenosis to occlusion of ICA, 3 patients (2.9%) were with isolated stenosis to occlusion of anterior cerebral artery (ACA).

**Table 2:** Baseline characterstics of LAA mild stroke patients with and without tandem lesions.

**Characteristics**	**Tandem lesions (*n* = 33)**	**No tandem lesions (*n* = 102)**	***P***
**Socio-demographic Characteristics**			
Female, *n* (%)	7 (21.2)	33 (32.3)	0.223
Age, y, mean ± SD	64.9 ± 13.2	63.8 ± 12.2	0.660
**Clinical status before admission**			
Previous stroke, *n* (%)	10 (30.3)	24 (23.5)	0.436
Hypertension, *n* (%)	21 (63.6)	73 (71.6)	0.389
Diabetes Mellitus, *n* (%)	15 (45.4)	24 (23.5)	0.016
Hyperlipidemia, *n* (%)	8 (24.2)	10 (9.80)	0.034
Smoking, *n* (%)	10 (30.3)	30 (29.4)	0.086
History of CHD, *n* (%)	0 (0.0)	10 (9.80)	0.118
**Clinical data on admission**			
SBP, mmHg, mean ± SD	158.3 ± 21.6	153.5 ± 24.4	0.319
DBP, mmHg, mean ± SD	88.5 ± 15.0	84.0 ± 14.8	0.131
TC, mmol/l, mean ± SD	4.1 ± 0.9	4.2 ± 1.0	0.539
TG, mmol/l, mean ± SD	1.8 ± 1.2	1.7 ± 1.1	0.667
LDL-C, mmol/l, mean ± SD	2.4 ± 0.7	2.5 ± 0.8	0.546
Cr, umol/l, mean ± SD	70.0 ± 14.4	72.4 ± 28.0	0.637
FPG, mmol/l, mean ± SD	6.8 ± 2.8	6.2 ± 4.6	0.486
NIHSS, mean ± SD	2.7 ± 1.4	2.6 ± 1.4	0.860
OTT time, min, mean ± SD	165.6 ± 46.9	160.5 ± 58.4	0.615

*LAA, large artery atherosclerosis; SD, standard deviation; CHD, coronary heart disease; SBP, systolic blood pressure; DBP, diastolic blood pressure; TC, total cholesterol; TG, triglycerides; LDL-C, low density lipoprotein cholesterol; Cr, creatinine; FPG, fasting blood glucose; NIHSS, National Institutes of Health Stroke Scale; OTT, onset-to-treatment*.

### Outcomes

**Figure 2:**
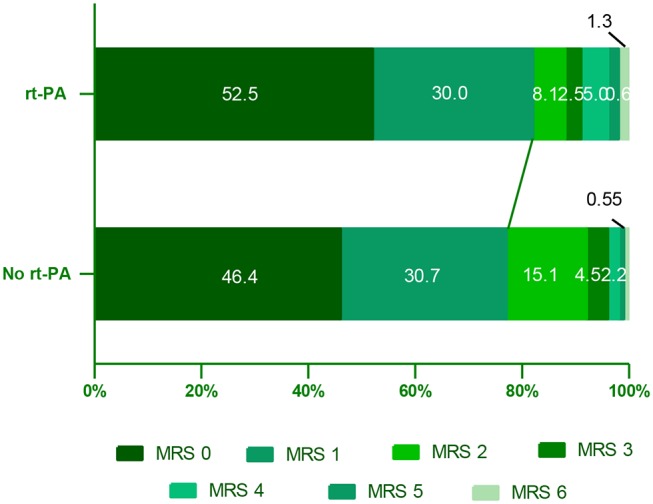
Distribution of mRS scores at 90 days in mild stroke patients with and without rt-PA treatment. mRS, modifed Rankin Scale.

In this study, we compared 90-day outcomes between mild stroke patients with and without rt-PA treatment. The mRS score distributions at this time point were shown for all mild stroke patients in Figure 2. We found that rt-PA-treated patients exhibited a non-significant trend toward more favorable outcomes relative to untreated patients after adjusting for age, sex, NIHSS score and OTT time (OR, 1.65; 95% CI, 0.96–2.86; *P* = 0.072), with 82.5% (132/160) in rt-PA treated group having good outcome (mRS: 0–1) at 90 days, while the proportion was 74.9% (134/179) in the untreated group.

**Figure 3:**
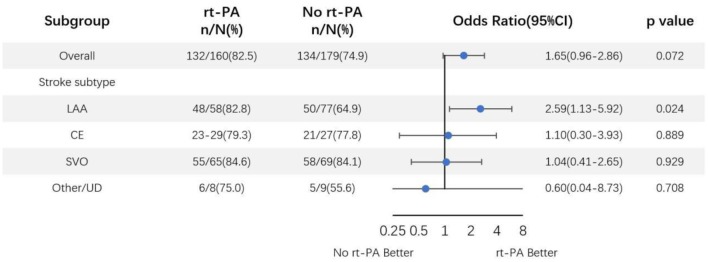
Odds ratio of rt-PA treatment for mild stroke patients after adjusting for age, sex, NIHSS score, and OTT time. CI, confidence; LAA, large artery atherosclerosis; CE, cardioembolism; SVO, small vessel occlusion; UD, undetermined.

We additionally conducted subgroup analyses according to stroke subtypes after adjusting for above specified covariates (Figure 3). This analysis revealed that LAA-type patients had significantly favorable outcomes at 90 days after rt-PA treatment compared to untreated patients (82.8 [48/58] vs. 64.9% [50/77]; OR, 2.59; 95% CI, 1.13–5.92; *P* = 0.024). In contrast, no such differences were observed in other stroke subtypes including cardioembolism (CE), small vessel occlusion (SVO), or other determined/undetermined subtypes (*P* = 0.889, *P* = 0.929, and 0.708, respectively). ORs and corresponding 95% CIs for patients in the rt-PA-treated and -untreated groups were shown in Figure 3.

**Figure 4:**
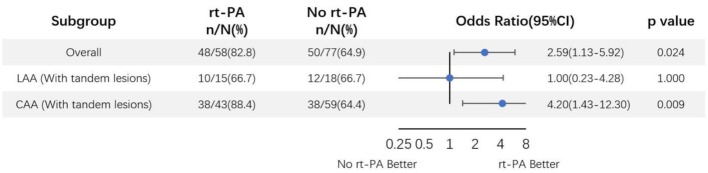
Odds ratio of rt-PA treatment for large-artery atherosclerosis mild stroke patients with and without tandem lesions after adjusting for age, sex, NIHSS score, and OTT time. CI, confidence; LAA, large artery atherosclerosis.

An additional subgroup analysis was performed adjusting for above specified covariates was performed wherein LAA-type patients were subdivided based upon whether or not they presented tandem lesions (Figure 4). In this analysis, we found that 66.7% (10/15) and 66.7% (12/18) of LAA tandem lesion patients in the rt-PA-treated and -untreated subgroups, respectively, exhibited favorable outcomes at the 90-day time point (OR, 1.00; 95%CI, 0.23–4.28; *P* = 1.000). Based on these analyses, we concluded that rt-PA treatment of LAA-type patients without tandem lesions was associated with a clear beneficial effect, with favorable outcome proportions of 88.4% (38/43) and 64.4% (38/59) in the treated and untreated groups, respectively (OR, 4.20; 95%CI, 1.43–12.30; *P* = 0.009).

Overall, we observed mortality rates of 1.2% (*n* = 2/160) and 0.6% (*n* = 1/178) among rt-PA-treated and -untreated patients, respectively (OR 2.25; 95%CI, 0.20–25.09; *P* = 0.604). Only two (1.2%) patients in the rt-PA treatment group exhibited symptomatic intracranial hemorrhage (sICH) within the 90-day study period, as determined based upon ECASSII criteria.

## Discussion

The results of the present study suggest that the IV rt-PA treatment of mild stroke patients is associated with high rates (82.5%) of 90-day favorable outcomes (mRS 0-1). In particular, we found that patients with LAA-subtype strokes were more likely to benefit from rt-PA treatment than patients suffering from other stroke subtypes. However, we found that the therapeutic benefits of rt-PA treatment in LAA patients were limited to patients that did not exhibit tandem steno-occlusion. In patients with such tandem lesions, these results instead suggest that the efficacy of IV rt-PA treatment may be limited.

In this study, we were able to estimate the effect of rt-PA in mild stroke patients who were excluded from most randomized trials based on varied exclusion criteria (24). Our findings also suggest that mild stroke patients may benefit from rt-PA treatment, particularly patients suffering from strokes with an LAA-type etiology. Previous randomized (25–28) and observational (29, 30) studies have yielded inconsistent findings with respect to the therapeutic benefits of rt-PA treatment in mild stroke patients. One prior pooled analysis of six thrombolysis trials failed to find any reason to exclude mild stroke patients from undergoing rt-PA treatment (31); these findings are consistent with findings from the NINDS rt-PA stroke subgroup study regarding minor stroke (32). One prospective cohort study found that, regardless of their baseline NIHSS scores, patients suffering from mild but with disabling symptoms should undergo rt-PA (33). In addition, one retrospective study of Chinese patients similarly found that patients with LAA-type strokes were likely to benefit from undergoing rt-PA treatment (3).

Treatment of AIS patients with tandem lesions is known to be technically challenging. However, there have been no specific studies conducted to date regarding the relative impact of rt-PA treatment in LAA-type mild stroke patients with and without tandem lesions. In the present study, we found that rt-PA treatment of mild stroke patients was associated with a therapeutic benefit only in LAA-subtype patients that did not exhibit tandem steno-occlusion. In LAA patients exhibiting such tandem lesions, our findings suggest that the benefits of rt-PA intervention may be minimal. All the following previous studies focused on the rt-PA treatment on tandem lesions were enrolled with severe stroke patients (NIHSS>5). Tandem lesions treated with rt-PA may sometimes achieve recanalization, but have poorer outcome than those with isolated occlusion (34–37). A large prospective study demonstrates that identification of a tandem lesion independently predicts poor response to rt-PA in terms of recanalization (38–40). While platelet activation is accelerated at the site of ruptured atherosclerotic plaque (18, 38), the lack of effect of rt-PA could be related to the fact that most tandem lesions were secondary to atherothrombotic disease of the carotid artery with large clot burden, which is a known predictor of poor recanalization after rt-PA (41–43). Kim et al. supposed that the low recanalization rate may be attributed to the large amount of thrombus (44). ICA lesion may cause hypoperfusion and reduce the delivery of rtPA to the thrombus in the MCA and ACA. Moreover, experimental researches demonstrated that the presence of a cervical ICA lesion (complete occlusion or severe stenosis) leads to decrease of cerebral perfusion pressure, which not only hinder dissolution of distal vessels but also increase the possibility of rethrombosis after incomplete recanalization (42). In terms of carotid atherothrombosis, the presence of a distal MCA occlusion usually is associated to an artery to artery embolism, with a clot proceeding from the carotid plaque which was platelet-rich lytic-resistant (41). Despite this, Ali et al. confirmed that 30% of the tandem -PAlesions achieved good outcome treated with rt-PA. AIS with tandem lesions should not be used as an exclusion from standard rt-PA therapy within the therapeutic window (18).

Based on the evidence of recent clinical trials, including HERMES (45), STRATIS (46), MR CLEAN (47), ESCAPE (48), and REVASCAT (49) trials have confirmed the benefit and favorable clinical outcomes of endovascular treatment for AIS with tandem lesions. A recent meta-analysis, data suggest that patients with tandem lesions can achieve spontaneous intracranial recanalization after recanalization of the cervical ICA (50, 51). Despite the obvious benefit from endovascular treatment for tandem lesions, there is no consensus about the ideal technical strategy (34, 45). Different endovascular approaches have been proposed; however, there has been no standardized recommendation (43). Previous studies comparing bridging therapy to mechanical thrombectomy (MT) alone demonstrated a higher rate of successful reperfusion and functional independence with bridging therapy. The results support the current guidelines of offering intravenous thrombolysis to eligible patients even if they are being considered for MT (52). TITAN (Thrombectomy in Tandem occlusions) which was a result of a collaborative effort to identify the best therapeutic approach for AIS due to tandem lesions (16), demonstrated that an approach associating emergent carotid stenting with rt-PA in conjunction to thrombectomy may be the best therapeutic option for AIS with tandem lesions leading to higher reperfusion and recanalization rates; this approach was not found to be associated with an increased risk of hemorrhagic complications (39, 53, 54).

Our study has strengths and limitations. The main strength is that we analyzed the effect on rt-PA treatment based on TOAST classification in mild stroke patients. Furthermore, in a subgroup analysis, we analyzed rt-PA treatment in mild stroke patients with tandem lesions which were with limited evidence for the optimal management. There are limitations to the present study. First, the population of patients in the subgroup of LAA-type patients with tandem lesions was quite small, our statistical power was therefore relatively limited. In addition, this study was retrospective and observational in nature, thus it does not offer robust insight regarding the clinical benefit of the IV rt-PA treatment of mild stroke patients. Future research efforts including scientifically rigorous randomized clinical trials will be needed in order to formally establish the clinical relevance of our findings.

## Conclusion

In summary, the results of this study indicate that intravenous rt-PA is only beneficial for the treatment of mild stroke patients with LAA-subtype strokes and without tandem steno-occlusion. Our findings suggest that intravenous rt-PA treatment may be of limited value in mild stroke patients with tandem lesions. The rapid vascular assessment and treatment of mild stroke patients are clinically important, as it allows for the identification of patients in need of emergency endovascular therapy.

## Data Availability Statement

The datasets generated for this study are available on request to the corresponding author.

## Ethics Statement

The studies involving human participants were reviewed and approved by The First Affiliated Hospital of Soochow University ethics committee approved the present study, with all relevant guidelines, and regulations being observed. The patients/participants provided their written informed consent to participate in this study. Written informed consent was obtained from the individual(s) for the publication of any potentially identifiable images or data included in this article.

## Author Contributions

DW and LZ conceived the research and wrote the main manuscript text. XT and DD participated in the recruitment of the sample population. XH and JZ acquired the data, analyzed the results. YK helped in interpreted the results and revised the article. XC, LL, and QF guided the process, interpreted the results, and revised the article. All authors read and approved the manuscript.

## Conflict of Interest

The authors declare that the research was conducted in the absence of any commercial or financial relationships that could be construed as a potential conflict of interest.
